# Supplementary material for: Analysis of Visuo Motor Control between Dominant Hand and Non-Dominant Hand for Effective Human-Robot Collaboration
Source: Sensors (Basel). 2020 Nov 8;20(21):6368. doi: 10.3390/s20216368 (PMC7664673; doi:10.3390/s20216368)
Supplement: Supplementary file 1 [file sensors-20-06368-s001.pdf]

sensors

MDPI

**Supplementary tables**

**Table S1.** A summary of the statistical analysis of $\Delta R$.

| Item | Variable | Test | Statistic | Confidence |
|---|---|---|---|---|
| A | $\Delta R$ between the dominant and non-dominant at each target speed | Two-way repeated measures ANOVA | Hand: Mauchly's Test $\chi^2(0) = 0$, $p$ = Nothing, $\varepsilon = 1$; <br> $F$ (1,1.436) = 4.585; <br><br> speed: Mauchly's Test $\chi^2(5) = 46.948$, $p = 0$, $\varepsilon = 0.488$; <br> $F$ (2.153,30.148) = 118.052; <br><br> interaction: Mauchly's Test $\chi^2(5) = 6.724$, $p = 0.242$, $\varepsilon = 0.942$; <br> $F$ (2.827,81.993) = 1.843 | Hand: $p = 0.041$, *partial* $\eta^2 = 0.137$, power = 0.544, corrected by Greenhouse-Geisser; <br><br> speed: $p = 0$, *partial* $\eta^2 = 0.803$, power = 1.0, corrected by Greenhouse-Geisser; <br><br> interaction: $p = 0.149$, *partial* $\eta^2 = 0.060$, power = 0.448, corrected by Huynh-Feldt |
| B | $\Delta R$ under the conditions of *V1*, *V2*, *V3*, *V4* between *dominant and non-dominant hand* | Bonferroni-corrected pairwise comparisons | *V1* between *dominant and non-dominant*: $t$ (29) = 0.331; <br><br> *V2* between *dominant and non-dominant*: $t$ (29) = 1.940; <br><br> *V3* between *Dominant and non-dominant*: $t$ (29) =2.286; <br><br> *V4* between *Dominant and non-dominant*: $t$ (29) = 1.807 | *V1* between *Dominant and non-dominant*: $p = 0.743$, *cohen's D = 0.061;* <br><br> *V2* between *Dominant and non-dominant*: $p = 0.062$, *cohen's D = 0.354;* <br><br> *V3* between *Dominant and non-dominant*: $p = 0.030$, *cohen's D = 0.417;* <br><br> *V4* between *Dominant and non-dominant*: $p = 0.081$, *cohen's D = 0.330;* |
| C | $\Delta R$ of target speeds under the conditions of *V1* : *V2*, | Bonferroni-corrected | *V1*: *V2*: $t$ *(29)* = 1.149; | *V1*: *V2*; $p = 1$, *cohen's D = 0.210;* |

| | | | | |
|---|---|---|---|---|
| | *V1* : *V3*, *V1* : *V4*, *V2* : *V3*, *V2* : *V4*, *V3* : *V4* in the dominant hand | pairwise comparisons | *V1*: *V3*: *t (29)* = 9.237;<br>*V1*: *V4*: *t (29)* = 8.554;<br><br>*V2*: *V3*: *t (29)* = 8.802;<br>*V2: V4*: *t (29)* = 8.195;<br><br>*V3*: *V4*: *t (29)* = 4.646 | *V1*: *V3*: *p* = 0, *cohen's D = 1.686;*<br>*V1*: *V4*: *p* = 0, *cohen's D = 1.562;*<br><br>*V2*: *V3*: *p* = 0, *cohen's D = 1.607;*<br>*V2: V4*: *p* = 0, *cohen's D = 1.496;*<br><br>*V3*: *V4*: *p* = 0, *cohen's D = 0.848* |
| D | Δ*R* of target speeds under the conditions of *V1* : *V2*, *V1* : *V3*, *V1* : *V4*, *V2* : *V3*, *V2* : *V4*, *V3* : *V4* in the non-dominant hand | Bonferroni-corrected pairwise comparisons | *V1*: *V2*: *t (29)* = 5.689;<br>*V1*: *V3*: *t (29)* = 14.643;<br>*V1*: *V4*: *t (29)* = 14.747;<br><br>*V2*: *V3*: *t (29)* = 9.063;<br>*V2*: *V4*: *t (29)* = 9.971;<br><br>*V3*: *V4*: *t (29)* = 5.336 | *V1*: *V2*: *p* = 0, *cohen's D = 1.039;*<br>*V1*: *V3*: *p* = 0, *cohen's D = 2.674;*<br>*V1*: *V4*: *p* = 0, *cohen's D = 2.692;*<br><br>*V2*: *V3*: *p* = 0, *cohen's D = 1.655;*<br>*V2*: *V4*: *p* = 0, *cohen's D = 1.820;*<br><br>*V3*: *V4*: *p* = 0, *cohen's D =0.974* |

**Table S2.** A summary of the statistical analysis of $\Delta\theta$.

| Item | Variable | Test | Statistic | Confidence |
|---|---|---|---|---|
| A | $\Delta\theta$ between the dominant and non-dominant at each target speed | Two-way repeated measures ANOVA | Hand: Mauchly's Test $\chi^2(0) = 0$, $p$ = Nothing, $\varepsilon = 1$; $F\ (1,29) = 27.291$; speed: Mauchly's Test $\chi^2(5) = 75.050$, $p = 0$, $\varepsilon = 0.463$; $F\ (1.389, 40.281) = 170.424$; interaction: Mauchly's Test $\chi^2(5) = 29.878$, $p = 0$, $\varepsilon = 0.644$; $F\ (1.933, 56.050) = 12.718$ | Hand: $p = 0$, *partial* $\eta^2 = 0.485$, power = 0.999, corrected by Greenhouse-Geisser; speed: $p = 0$, *partial* $\eta^2 = 0.855$, power = 1, corrected by Greenhouse-Geisser; interaction: $p = 0$, *partial* $\eta^2 = 0.305$, power = 0.994, corrected by Greenhouse-Geisser |
| B | $\Delta\theta$ under the conditions of *V1*, *V2*, *V3*, *V4* between *dominant and non-dominant hand* | Bonferroni-corrected pairwise comparisons | *V1* between *dominant and non-dominant*: $t\ (29) = 0.153$; *V2* between *dominant and non-dominant*: $t\ (29) = 2.043$; *V3* between *Dominant and non-dominant*: $t\ (29) = 4.775$; *V4* between *Dominant and non-dominant*: $t\ (29) = 4.543$ | *V1* between *Dominant and non-dominant*: $p = 0.879$, *cohen's D = 0.028;* *V2* between *Dominant and non-dominant*: $p = 0.050$, *cohen's D = 0.373;* *V3* between *Dominant and non-dominant*: $p = 0$, *cohen's D = 0.872* *V4* between *Dominant and non-dominant*: $p = 0$, *cohen's D = 0.830;* |
| C | $\Delta\theta$ of target speeds under the conditions of *V1* : *V2*, *V1* : *V3*, *V1* : *V4*, *V2* : *V3*, *V2* | Bonferroni-corrected pairwise comparisons | *V1*: *V2*: $t\ (29) = 4.383$; *V1*: *V3*: $t\ (29) = 8.850$; | *V1*: *V2*; $p = 0.001$, *cohen's D = 0.800;* *V1*: *V3*: $p = 0$, *cohen's D = 1.616;* |

| | | | | |
|---|---|---|---|---|
| | : *V4*, *V3* : *V4* in the dominant hand | | *V1*: *V4*: *t (29)* = 13.378;<br>*V2*: *V3*: *t (29)* = 8.333;<br>*V2*: *V4*: *t (29)* = 12.947;<br>*V3*: *V4*: *t (29)* = 11.217 | *V1*: *V4*: *p* = 0, *cohen's D = 2.443;*<br>*V2*: *V3*: *p* = 0, *cohen's D = 1.521;*<br>*V2*: *V4*: *p* = 0, *cohen's D = 2.364;*<br>*V3*: *V4*: *p* = 0, *cohen's D = 2.048* |
| D | $\Delta\theta$ of target speeds under the conditions of *V1* : *V2*, *V1* : *V3*, *V1* : *V4*, *V2* : *V3*, *V2* : *V4*, *V3* : *V4* in the non-dominant hand | Bonferroni-corrected pairwise comparisons | *V1*: *V2*: *t (29)* = 8.004;<br>*V1*: *V3*: *t (29)* = 11.971;<br>*V1*: *V4*: *t (29)* = 13.852;<br>*V2*: *V3*: *t (29)* = 9.053;<br>*V2*: *V4*: *t (29)* = 13.086;<br>*V3*: *V4*: *t (29)* = 9.341 | *V1*: *V2*: *p* = 0, *cohen's D = 1.461;*<br>*V1*: *V3*: *p* = 0, *cohen's D = 2.186;*<br>*V1*: *V4*: *p* = 0, *cohen's D = 2.529;*<br>*V2*: *V3*: *p* = 0, *cohen's D = 1.653;*<br>*V2*: *V4*: *p* = 0, *cohen's D = 2.389;*<br>*V3*: *V4*: *p* = 0, *cohen's D =1.705* |

**Table S3.** A summary of the statistical analysis of $\Delta a$.

| Item | Variable | Test | Statistic | Confidence |
|---|---|---|---|---|
| A | $\Delta a$ between the dominant and non-dominant at each target speed | Two-way repeated measures ANOVA | Hand: Mauchly's Test$\chi^2(0) = 0$, *p* = Nothing, $\varepsilon = 1$;<br>*F* (1,29) = 11.381;<br>speed: Mauchly's Test$\chi^2(5) = 113.933$, $p = 0$, $\varepsilon = 0.386$;<br>*F* (1.157, 33.564) = 248.442;<br>interaction: Mauchly's Test$\chi^2(5) = 56.727$, $p = 0$, $\varepsilon = 0.538$;<br>*F* (1.615, 46.848) = 3.474 | Hand: $p = 0.002$, *partial* $\eta^2 = 0.282$, power = 0.903, corrected by Greenhouse-Geisser;<br>speed: $p = 0$, *partial* $\eta^2 = 0.895$, power = 1, corrected by Greenhouse-Geisser;<br>interaction: $p = 0.049$, *partial* $\eta^2 = 0.107$, power = 0.562, |

| | | | | |
|---|---|---|---|---|
| | | | | corrected by Greenhouse-Geisser |
| B | $\Delta\alpha$ under the conditions of *V1, V2, V3, V4* between *dominant and non-dominant hand* | Bonferroni-corrected pairwise comparisons | *V1* between *dominant and non-dominant*: *t* (29) = 2.760;<br><br>*V2* between *dominant and non-dominant*: *t* (29) = 2.502;<br><br>*V3* between *Dominant and non-dominant*: *t* (29) =3.659;<br><br>*V4* between *Dominant and non-dominant*: *t* (29) = 2.070 | *V1* between *Dominant and non-dominant*: *p* = 0.010, *cohen's D = 0.504;*<br><br>*V2* between *Dominant and non-dominant*: *p* = 0.018, *cohen's D = 0.457;*<br><br>*V3* between *Dominant and non-dominant*: *p* = 0.001, *cohen's D = 0.668;*<br><br>*V4* between *Dominant and non-dominant*: *p* = 0.047, *cohen's D = 0.378* |
| C | $\Delta\alpha$ of target speeds under the conditions of *V1* : *V2*, *V1* : *V3*, *V1* : *V4*, *V2* : *V3*, *V2* : *V4*, *V3* : *V4* in the dominant hand | Bonferroni-corrected pairwise comparisons | *V1*: *V2*: *t (29)* = 13.296;<br>*V1*: *V3*: *t (29)* = 18.789;<br>*V1*: *V4*: *t (29)* = 17.435;<br><br>*V2*: *V3*: *t (29)* = 15.893;<br>*V2*: *V4*: *t (29)* = 15.993;<br><br>*V3*: *V4*: *t (29)* = 11.689 | *V1*: *V2*; *p* = 0, *cohen's D = 2.428;*<br>*V1*: *V3*: *p* = 0, *cohen's D = 3.430;*<br>*V1*: *V4*: *p* = 0, *cohen's D = 3.183;*<br><br>*V2*: *V3*: *p* = 0, *cohen's D = 2.902;*<br>*V2*: *V4*: *p* = 0, *cohen's D = 2.920;*<br><br>*V3*: *V4*: *p* = 0, *cohen's D = 2.134* |
| D | $\Delta\alpha$ of target speeds under the conditions of *V1* : *V2*, *V1* : *V3*, *V1* : *V4*, *V2* : *V3*, *V2* : *V4*, *V3* : *V4* in the non-dominant hand | Bonferroni-corrected pairwise comparisons | *V1*: *V2*: *t (29)* = 13.005;<br>*V1*: *V3*: *t (29)* = 13.721;<br>*V1*: *V4*: *t (29)* = 13.787;<br><br>*V2*: *V3*: *t (29)* = 11.516; | *V1*: *V2*: *p* = 0, *cohen's D = 2.374;*<br>*V1*: *V3*: *p* = 0, *cohen's D = 2.505;*<br>*V1*: *V4*: *p* = 0, *cohen's D = 2.517;*<br><br>*V2*: *V3*: *p* = 0, *cohen's D = 2.103;* |

| | | | |
|---|---|---|---|
| | | *V2*: *V4*: *t (29)* = 12.610; | *V2*: *V4*: *p* = 0, *cohen's D = 2.302;* |
| | | *V3*: *V4*: *t (29)* = 10.178; | *V3*: *V4*: *p* = 0, *cohen's D =1.858* |

**Table S4.** A summary of the statistical analysis of *DSJ*(Dimensionless Squared Jerk).

| Item | Variable | Test | Statistic | Confidence |
|---|---|---|---|---|
| A | Dimensionless Squared Jerk between the dominant and non-dominant at each target speed | Two-way repeated measures ANOVA | Hand: Mauchly's Test$\chi^2(0) = 0$, $p$ = Nothing, $\varepsilon = 1$;<br><br>$F$ (1,29) = 20.868;<br><br>speed: Mauchly's Test$\chi^2(5) = 547.565$, $p = 0$, $\varepsilon = 0.334$;<br><br>$F$ (1.002, 29.047) = 85.913;<br><br>interaction: Mauchly's Test$\chi^2(5) = 587.401$, $p = 0$, $\varepsilon = 0.334$;<br><br>$F$ (1.002, 29.047) = 20.156 | Hand: $p = 0$, *partial* $\eta^2 = 0.418$, power = 0.993, corrected by Greenhouse-Geisser;<br><br>speed: $p = 0$, *partial* $\eta^2 = 0.748$, power = 1, corrected by Greenhouse-Geisser;<br><br>interaction: $p = 0$, *partial* $\eta^2 = 0.410$, power = 0.991, corrected by Greenhouse-Geisser |
| B | Dimensionless Squared Jerk under the conditions of *V1*, *V2*, *V3*, *V4* between *dominant and non-dominant hand* | Bonferroni-corrected pairwise comparisons | *V1* between *dominant and non-dominant*: $t$ (29) = 4.511;<br><br>*V2* between *dominant and non-dominant*: $t$ (29) = 3.377;<br><br>*V3* between *Dominant and non-dominant*: $t$ (29) =0.050;<br><br>*V4* between *Dominant and non-dominant*: $t$ (29) = 0.054 | *V1* between *Dominant and non-dominant*: $p = 0$, *cohen's D = 0.824*<br><br>*V2* between *Dominant and non-dominant*: $p = 0.002$, *cohen's D = 0.617;*<br><br>*V3* between *Dominant and non-dominant*: $p = 0.961$, *cohen's D = 0.009;*<br><br>*V4* between *Dominant and non-dominant*: $p = 0.957$, *cohen's D = 0.010* |

| | | | | |
|---|---|---|---|---|
| C | Dimensionless Squared Jerk of target speeds under the conditions of *V1* : *V2*, *V1* : *V3*, *V1* : *V4*, *V2* : *V3*, *V2* : *V4*, *V3* : *V4* in the dominant hand | Bonferroni-corrected pairwise comparisons | *V1*: *V2*: *t (29)* = 6.911; *V1*: *V3*: *t (29)* = 7.007; *V1*: *V4*: *t (29)* = 7.009; *V2*: *V3*: *t (29)* = 5.321; *V2*: *V4*: *t (29)* = 5.381 *V3*: *V4*: *t (29)* = 10.652; | *V1*: *V2*; *p* = 0, *cohen's D* = *1.262;* *V1*: *V3*: *p* = 0, *cohen's D* = *1.279;* *V1*: *V4*: *p* = 0, *cohen's D* = *1.280;* *V2*: *V3*: *p* = 0, *cohen's D* = *0.971;* *V2: V4*: *p* = 0, *cohen's D* = *0.982;* *V3*: *V4*: *p* = 0, *cohen's D = 1.945* |
| D | Dimensionless Squared Jerk of target speeds under the conditions of *V1* : *V2*, *V1* : *V3*, *V1* : *V4*, *V2* : *V3*, *V2* : *V4*, *V3* : *V4* in the non-dominant hand | Bonferroni-corrected pairwise comparisons | *V1 V2*: *t (29)* = 21.279; *V1*: *V3*: *t (29)* = 21.190; *V1*: *V4*: *t (29)* = 21.173; *V2*: *V3*: *t (29)* = 15.341; *V2*: *V4*: *t (29)* = 15.034 *V3*: *V4*: *t (29)* = 9.026; | *V1*: *V2*: *p* = 0, *cohen's D* = *3.885;* *V1*: *V3*: *p* = 0, co*hen's D* = *3.869;* *V1*: *V4*: *p* = 0, *cohen's D* = *3.866;* *V2*: *V3*: *p* = 0, *cohen's D* = *2.801;* *V2*: *V4*: *p* = 0, *cohen's D* = *2.745;* *V3*: *V4*: *p* = 0, *cohen's D =1.648* |
